# Cytotoxic Activities of Flavonoids from *Centaurea scoparia*

**DOI:** 10.1155/2014/274207

**Published:** 2014-06-11

**Authors:** Sayed A. Ahmed, Emadeldin M. Kamel

**Affiliations:** Chemistry Department, Faculty of Science, Beni Suef University, Salah Salem Street, P.O. Box 62514, Beni Suef 62514, Egypt

## Abstract

Phytochemical studies on the ethanolic extract of the aerial parts of *Centaurea scoparia* led to the isolation of two new flavonoids, 3′,4′-dihydroxy-(3′′,4′′-dihydro-3′′-hydroxy-4′′-acetoxy)-2′′,2′′-dimethylpyrano-(5′′,6′′:7,8)-flavone-3-*O*-**β**-D-glucopyranoside (**1**) and 3,3′,4′-trihydroxy-(3′′,4′′-dihydro-3′′,4′′-dihydroxy)-2′′,2′′-dimethylpyrano-(5′′,6′′:7,8)-flavone (**2**), along with eight known flavonoids isolated for the first time from this plant, cynaroside (**3**), Apigetrin (**4**), centaureidin (**5**), oroxylin A (**6**), 5,7-dihydroxy-3′,4′,5′-trimethoxyflavone (**7**), atalantoflavone (**8**), 5-hydroxy-3′,4′,8-trimethoxy-2′′,2′′-dimethylpyrano (5′′,6′′:6,7)-flavone (**9**), and 3′,4′,5,8-tetramethoxy-2′′,2′′-dimethylpyrano (5′′,6′′:6,7)-flavone (**10**). The structures of the isolated compounds were elucidated by means of spectroscopic tools including 1D and 2D NMR, UV, IR, and mass spectroscopy. Cytotoxic activities of the isolated compounds were evaluated against human cervical carcinoma HeLa, human hepatocellular carcinoma HepG2, and human breast carcinoma MCF-7. Compound **2** was the most potent cytotoxic agent against HeLa cells with an IC_50_ 0.079 **μ**M.

## 1. Introduction

The genus* Centaurea* belongs to the family Asteraceae and contains several hundreds of species of herbaceous thistle which are mainly distributed north of the equator, mostly in the Middle East and Western Asia [1]. Many of the* Centaurea* species have been utilized in traditional medicine for the treatment of cancer [2], microbial infections [3, 4] and as stimulant, tonic [5, 6], antidiabetic [7–9], diuretic [7], and antirheumatic [10, 11].* C. scoparia* is an annual or biannual herb growing in Sinai, Egypt [11]. Previous phytochemical screening showed that* C. scoparia* contains phenolic compounds including luteolin, apigenin, hispidulin, cirsimaritin, salvigenin, (-)-matairesinol, (-)-arctigenin, vanillin, and omega-hydroxypropioguaiacone [12]. Also,* C. scoparia* contains chlorinated and nonchlorinated guaianolides including diain, janerin, cynaropicrin, deacylcynaropicrin [13, 14], and other sesquiterpenes lactones [15, 16]. In our research on phenolic constituents and biological activities of folk medicinal plants, we have found that the ethanolic extract of the aerial parts* C. scoparia* exhibited a potent cytotoxic activity against human cancer cell lines. So a phytochemical fractionation for the ethanolic extract of* C. scoparia* has been performed and led to the isolation of two new hydropyranoflavones(**1**,** 2**), two known flavonoid glycosides(**3**,** 4**), three known flavonoid aglycones (**5**–**7**), and three known pyranoflavones (**8**–**10**).

## 2. Experimental

### 2.1. General

^1^H (500 MHz) and ^13^C (125 MHz) NMR were recorded in DMSO-*d*_6_ on a Bruker AV-500 NMR spectrometer using TMS as internal standard. Chemical shifts (*δ*) are expressed in ppm and coupling constants (*J*) are reported in Hz. Optical rotations were measured with Perkin-Elmer 341 polarimeter. UV data were obtained from Shimadzu UV-vis 160i spectrophotometer. HREIMS and EIMS mass spectroscopic analysis was measured on a Finnigan MAT TSQ 700 mass spectrometer. IR spectra were recorded on KBr pellets on a Shimadzu FTIR-8400 instrument.

### 2.2. Plant Material

The aerial parts of* C. scoparia* were collected from the Eastern desert close to Beni Suef governorate on April 2011. Identification of the plant was confirmed by the Department of flora, Agricultural Museum, Ministry of Agriculture and Herbarium of the Department of Botany, Faculty of Science, Cairo University. Voucher specimen was kept in Herbarium, National Research Center, Giza, Egypt.

### 2.3. Extraction and Isolation

The dried and powdered aerial parts of* C. scoparia* (5 kg) were extracted at room temperature with 70% EtOH (10 L × 4 times). The solvent in the combined extracts was removed under reduced pressure at temperature not exceeding 40°C till dryness to give a crude residue (275 g) which was successively partitioned with hexane (1 L × 3 times), ethyl acetate (1 L × 3 times), and *n*-butanol (1 L × 3 times) to give three extracts hexane (70 g), ethyl acetate (50 g), and *n*-butanol (75 g). The ethyl acetate extract was subjected to fractionation on a column (150 × 4 cm) containing 600 gm of polyamide 6S. Gradient elution started with water followed by H_2_O/EtOH mixtures of decreasing polarities (10 : 0, 9 : 1, 8 : 2, 7 : 3, 6 : 4, 5 : 5, 4 : 6, 3 : 7, 2 : 8, 1 : 9, and 0 : 10). The bands migrated along the column were observed under UV light during elution process to control the fractionation process as possible. A total of 44 fractions were collected and combined into 12 main fractions (E1–E12) according to their TLC behavior. These fractions were dried under* Vacou *≈40°C and subjected to detailed investigations by TDPC and TLC. Phytochemical investigation of fractions E1 and E2 revealed the presence of trace amounts of phenolic compounds and majority of free sugars, which were detected by comparative paper chromatography. Fraction E3 (1.7 g) was applied to a polyamide column (35 × 2.5 cm) using EtOH/H_2_O mixture (1 : 1) as an eluent to give four subfractions (E3-1, E3-2, E3-3, and E3-4). Subfractions E3-2 and E3-3 were combined and purified on Sephadex LH-20 (30 × 1.5 cm) column and eluted with MeOH/H_2_O (1 : 1) to give the purified compounds** 1** (25 mg) and** 2** (32 mg). Fraction E4 (1.5 g) was chromatographed on Sephadex LH-20 column eluted by EtOH to give two major subfractions (E4-1 and E4-2). Subfraction E4-1 was further purified by ODS CC eluted with a gradient MeOH/H_2_O solvent system (from 5 : 5 to 9 : 1) to yield compound** 3** (21 mg). Subfraction E4-2 was further purified on Sephadex LH-20 column eluted by MeOH to give compound** 4** (19 mg). Fraction E8 (1.8 g) was rechromatographed on polyamide column (35 × 2.5 cm) using EtOH/H_2_O (8 : 2) to afford three major subfractions (E8-1, E8-2, and E8-3), and these subfractions were purified on Sephadex LH-20 columns using MeOH as an eluent to yield the purified compounds** 5** (27 mg),** 6** (17 mg), and** 7** (24 mg). Compounds** 8** (14 mg),** 9** (22 mg), and** 10** (18 mg) were obtained from fraction E9 (750 mg) after application to Sephadex LH-20 columns using MeOH/H_2_O (9.5 : 0.5) as an eluent to yield three major successive subfractions (E9-1, E9-2, and E9-3). Subfraction E9-1 was purified on Sephadex LH-20 columns using MeOH as an eluent to afford compound** 8**. Subfractions E9-2 and E9-3 were further purified by ODS CC using MeOH/H_2_O (8 : 2) to yield compounds** 9** and** 10**.

### 2.4. Cell Culture

All cell lines were purchased from the American Type Culture Collection (ATCC). Three cell lines were used in this study: HepG2 cells (human cell line of a well-differentiated hepatocellular carcinoma isolated from a liver), HeLa (cervical carcinoma cells), and MCF-7 (breast carcinoma cells). Cells were grown or maintained upon arrival at 37°C in a humidified incubator with 5% CO_2_ and 95% atmosphere as recommended by ATCC. All media were supplemented with penicillin (100 U/mL), streptomycin (100 *μ*g/mL), and 10% heat-inactivated fetal bovine serum (FBS).

### 2.5. Cytotoxicity Assay

Cytotoxicity was quantitatively evaluated by the 3-(4,5-dimethylthiazol-2-yl)-2,5-diphenyl tetrazolium bromide (MTT) method as previously described [17]. The cells were seeded in 96-well plate at a cell concentration of 1 × 10^4^ cells per well in 100 µL of growth medium then treated with various concentrations of test compounds and incubated in a humidified 5% CO_2_ atmosphere at 37°C for 72 h. After the incubation, 10 *μ*L of 5 mg/mL MTT was added to each well and incubated for another 4 h. After removal of the supernatant, formazan crystals were dissolved in 100 *μ*L DMSO and the optical density values were measured at 570 nm with a microplate reader. Deguelin was used as the positive control.

## 3. Results and Discussion

**Figure 1:**
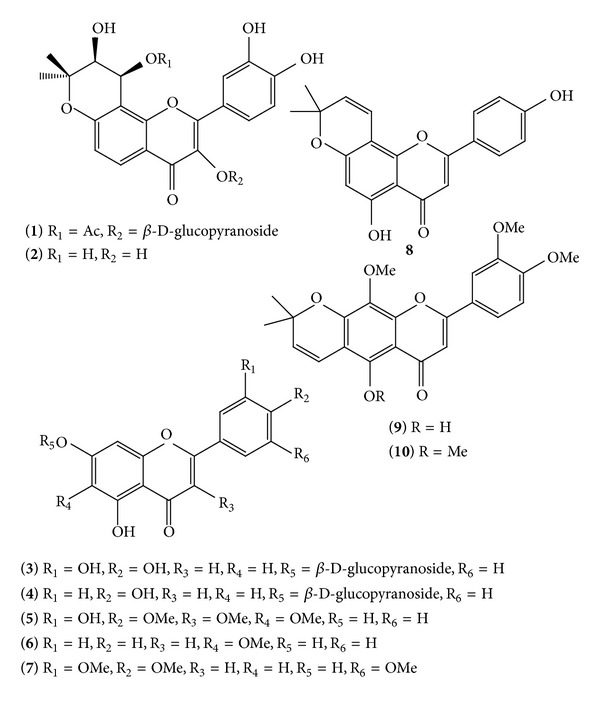
Structure of compounds** 1**–**10** isolated from* C. scoparia*.

A phytochemical investigation of the ethyl acetate extract of* C. scoparia *aerial parts led to the isolation of two new (**1**-**2**) and eight known (**3**–**10**) flavonoids isolated for the first time from this plant (Figure 1). The structures of the new compounds 3′,4′-dihydroxy-(3′′,4′′-dihydro-3′′-hydroxy-4′′-acetoxy)-2′′,2′′-dimethylpyrano-(5′′,6′′:7,8)-flavone-3-*O*-*β*-D-gulcopyranoside (**1**) and 3,3′,4′-trihydroxy-(3′′,4′′-dihydro-3′′,4′′-dihydroxy)-2′′,2′′-dimethylpyrano-(5′′,6′′:7,8)-flavone (**2**) were elucidated by means of spectroscopic tools and the known compounds were identified as cynaroside (**3**) [18], Apigetrin (**4**) [19], centaureidin (**5**) [20, 21], oroxylin A (**6**) [22, 23], 5,7-dihydroxy-3′,4′,5′-trimethoxyflavone (**7**) [24, 25], atalantoflavone (**8**) [26], 5-hydroxy-3′,4′,8-trimethoxy-2′′,2′′-dimethylpyrano (5′′,6′′:6,7)-flavone (**9**) [27, 28], and 3′,4′,5,8-tetramethoxy-2′′,2′′-dimethylpyrano (5′′,6′′:6,7)-flavone (**10**) [27, 28].

**Figure 2:**
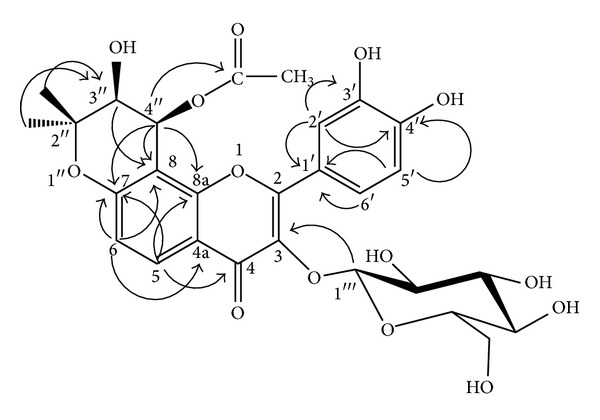
Representative HMBC correlations of compound** 1**.

Compound** 1** was obtained as a pale yellow amorphous powder. The UV spectrum showed maxima at 258 and 335 nm, suggesting a flavone moiety [28]. The IR spectrum displayed strong bands for OH (3371 cm^−1^) and conjugated carbonyl (1691 cm^−1^). The HREIMS mass spectrum revealed a [M + H]^+^ ion at *m*/*z* 590.5124 corresponding to the molecular formula C_28_H_30_O_14_. The ^1^H and ^13^C NMR spectrum of** 1** (Table 1), recorded in DMSO-*d*_6_, showed a downfield conjugated carbonyl *δ*_C_ 178.2 (C-4) and displayed a pair of aromatic protons at *δ*_H_ 7.51 and 6.95 (1H, *d*, *J* = 8.9 Hz each). The *J* values of 8.9 Hz indicated an* ortho*-coupling, hence diagnostic for protons at positions C-5 and C-6, respectively. The ^1^H NMR spectrum exhibited signals in the aromatic region at *δ*_H_ 7.11 (1H, *d*, *J* = 1.8 Hz), 7.01 (1H, *dd*, *J* = 8.1, 1.8 Hz), and 6.90 (1H, *d*, *J* = 8.1 Hz), which indicated a 1,3,4-trisubstituted phenyl group. The presence of a pair oxygenated methines at (*δ*_H_ 3.95, 1H, *d*, *J* = 4.8, H-3′′; *δ*_C_ 69.7, C-3′′) and (*δ*_H_ 6.55, 1H, *d*, *J* = 4.8, H-4′′; *δ*_C_ 63.5, C-4′′) indicated that an oxygen atom at C-7 was cyclized with C-2′′ to form a dioxo-substituted pyran ring. The presence of an acetoxy group was detected by a signal in the ^1^H NMR at *δ*_H_ 1.95 (3H, s) and two signals in the ^13^C NMR at *δ*_C_ 171 and *δ*_C_ 20.7 (OAc-4′′). Two methyl groups were detected from signals at *δ*_H_ 1.42 and 1.39 (3H, s each) for Me^1^-2′′ and Me^2^-2′′, respectively, confirmed by two signals in the ^13^C NMR at *δ*_C_ 26.6 (Me^1^-2′′) and 21.5 (Me^2^-2′′). An oxygenated quaternary carbon (C-2′′) was detected by a signal at *δ*_C_ 79.6. Searching the literature, we have found that compound** 1** was similar to 5-methoxy-(3′′,4′′-dihydro-3′′,4′′-diacetoxy)-2′′,2′′-dimethylpyrano-(7,8:5′′,6′′)-flavone [29]. By comparison of the NMR Spectral data of the two compounds, the differences were the presence of only one acetoxy group in compound** 1**, two* ortho*-coupled protons at C-5 and C-6, trisubstituted B-ring in compound** 1** as previously referred to, and the presence of a sugar moiety which was indicated by the signals of the anomeric proton at *δ*_H_ 4.95 (1H, *d*, *J* = 6.9) and *δ*_C_ 102.4, this coupling constant diagnostic for the *β*-anomer. We have observed an upfield shift in the *δ*_C_ of C-3 and a downfield shift in that of C-2 and C-4 which is diagnostic for the linkage of the sugar moiety to C-3. In the ^1^H NMR spectrum, signals at *δ*_H_ 4.95 (1H, *d*, *J* = 6.9); *δ*_H_ 3.11–3.45 (m) for the rest of the sugar protons and in the ^13^C NMR spectrum the signals at *δ*_C_ 102.4, 74.8, 77.9, 70.1, 76.7, and 61.3 suggesting the sugar to be *β*-glucopyranose. Furthermore, the sugar moiety was confirmed to be D-glucose by TLC analysis and coPC after hydrolysis. Besides the molish test the ESIMS fragment ions at 428 [M + H-162]^+^ also indicated the presence of a sugar moiety. The presence of a hydroxyl group at C-3′′ was indicated from the chemical shift in the ^13^C NMR *δ*_C_ 69.7 (C-3′′) and the HREIMS data. The 1D NMR data suggests that compound** 1** is a flavone with acetylated pyran ring and a sugar moiety. In the HMBC correlation as illustrated in Figure 2 and tabulated in Table 1, the signal at *δ*_H_ 7.51 (1H, *d*, *J* = 8.9 Hz, H-5) indicated three key correlations with carbon signals at *δ*_C_ 178.2 (C-4), 158.6 (C-7), and 155.7 (C-8a), the correlation between *δ*_H_ 7.51 (H-5) and *δ*_C_ 178.2 (C-4) indicated that the A-ring was unsubstituted at C-5 and C-6 positions. The proton signals at *δ*_H_ 6.95 (1H, *d*, *J* = 8.9 Hz, H-6) correlated with two quaternary carbons at *δ*_C_ 118.9 (C-4a) and *δ*_C_ 108.4 (C-8) as well as one oxygenated carbon at *δ*_C_ 158.6 (C-7). The correlation from the signal of H-3′′ (*δ*_H_ 3.95, 1H, *d*, *J* = 4.8) to the signal of C-8 (*δ*_C_ 108.4) and from the signal of H-4′′ (*δ*_H_ 6.55, 1H, *d*, *J* = 4.8) to the signal of C-8 (*δ*_C_ 108.4), C-7 (*δ*_C_ 158.6), and C-8a (*δ*_C_ 155.7) suggests that the dihydropyran ring is attached to the A-ring at C-8 and C-7 as a product of the cyclization of an oxygen at C-7 with C-2′′. The correlation between the signals of the proton of the two methyl groups (*δ*_H_ 1.39 and 1.42; 3H, s each) at C-2′′ with C-3′′ and that of H-4′′ (*δ*_H_ 6.55) with the carbonyl of the acetoxy group is a proof for the location of the acetoxy group to be at position C-4′′. The configuration of C-3′′ and C-4′′ was determined to be* cis* based on the value of the coupling constant (*J*_3′′,4′′_ = 4.8) and the difference in the values of the chemical shifts of the 2′′-*geminal* dimethyl groups (0.03 ppm) [29]. The HMBC correlation was further utilized to identify the linkage between the aglycone and sugar moiety. The correlation between H-1′′′ (*δ*_H_ 4.95, glc) and C-3 (*δ*_C_ 133.7) is a confirmation for the linkage of the sugar moiety to C-3 of the aglycone. Based on these assignments, the structure of compound** 1** was elucidated as 3′,4′-dihydroxy-(3′′,4′′-dihydro-3′′-hydroxy-4′′-acetoxy)-2′′,2′′-dimethylpyrano-(5′′,6′′:7,8)-flavone-3-*O*-*β*-D-gulcopyranoside.

**Figure 3:**
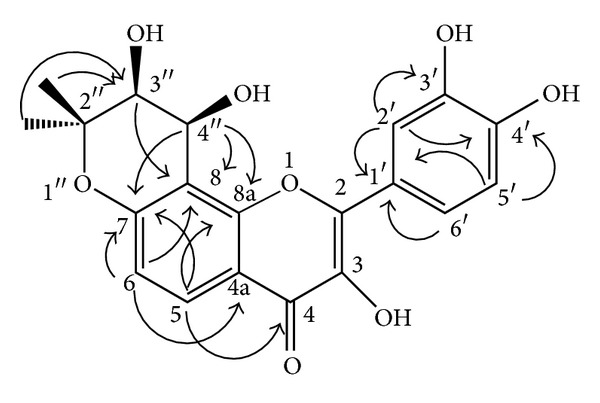
Representative HMBC correlation of compound **2**.

Compound** 2** was obtained as a white amorphous powder. It was shown to be closely related to compound** 1**. The IR spectrum showed strong bands for OH (3385 cm^−1^) and conjugated carbonyl (1695 cm^−1^). The UV spectrum of this compound also showed features of a flavone moiety (259 and 342 nm). The addition of shift reagents confirms the structure to be a flavone with* ortho*-dihydroxy group at positions 3′ and 4′. The HREIMS mass spectrum of** 2** revealed a [M + H]^+^ ion at *m*/*z* 386.4135 corresponding to the molecular formula C_20_H_18_O_8_. The NMR data of compound** 2** was very closely related to compound** 1** (Table 1), with the differences being the absence of the signals of the acetoxy group and the sugar moiety (negative molish test). The 1D NMR data of** 2** (Table 1), recorded in DMSO-*d*_6_, showed a signal for the conjugated carbonyl carbon (C-4) at a chemical shift lower than that of compound** 1** (*δ*_C_ 173.7), which is suggestion for the absence of the sugar moiety at C-3. The ^1^H NMR spectrum exhibited two signals at *δ*_H_ 7.55 and 6.97 (1H, *d*, *J* = 8.9 Hz each), diagnostic for the* ortho*-coupled protons at C-5 and C-6, respectively. The B-ring was assigned to be disubstituted from the signals in the aromatic region at *δ*_H_ 7.13 (1H, *d*, *J* = 1.8 Hz), 7.05 (1H, *dd*, *J* = 8.1, 1.8 Hz), and 6.93 (1H, *d*, *J* = 8.1 Hz). Two oxygenated methines were detected at (*δ*_H_ 3.81, 1H, *d*, *J* = 4.8, H-3′′; *δ*_C_ 69.4, C-3′′) and (*δ*_H_ 5.24, 1H, *d*, *J* = 4.8, H-4′′; *δ*_C_ 59.3, C-4′′), which is diagnostic for the cyclization of the oxygen atom at C-7 with the C-2′′ to form the dihydroxy substituted pyran ring. The value of the chemical shift of the proton at C-4′′ in compound** 1** is higher than that in compound** 2** due to the replacement of the acetoxy group in** 1** with a hydroxy group in** 2** which makes this proton more deshielded in** 1** than in** 2**. The two methyl groups at C-2′′ were confirmed by signals at (*δ*_H_ 1.41, s, 3H; *δ*_C_ 24.1, Me^1^-2′′) and (*δ*_H_ 1.38, s, 3H; *δ*_C_ 21.2, Me^2^-2′′). An oxygenated quaternary carbon at C-2′′ was assigned from a signal at *δ*_C_ 78.3. A confirmation for the absence of the sugar moiety at C-3 is the higher *δ*_C_ value of C-3 in compound** 1** (*δ*_C_ 133.7) than in** 2** (*δ*_C_ 141.2) and the decreasing of the *δ*_C_ of C-2 and C-4 to be 154.2 and 173.7, respectively. In the HMBC correlation (Table 1; Figure 3), the H-5 (*δ*_H_ 7.55) proton correlated with C-4 (173.7), C-7 (158.2), and C-8a (155.1) diagnostic for* ortho*-coupled aromatic protons at C-5 and C-6, confirmed by the proton signals at *δ*_H_ 6.97 (H-6) correlated to *δ*_C_ 117.8 (C-4a), *δ*_C_ 107.7 (C-8), and *δ*_C_ 158.2 (C-7). The attachment of the pyran ring to C-7 and C-8 was estimated from the correlation between the signal of H-3′′ (*δ*_H_ 3.81) with C-8 (107.7) and the signal of H-4′′ (*δ*_H_ 5.24) with C-8 (*δ*_C_ 107.7), C-7 (*δ*_C_ 158.2), and C-8a (*δ*_C_ 155.1). In comparing the HMBC correlation of compound** 1** and compound** 2**, we have found that the correlation of H-1′′′ with C-3 and H-4′′ with the carbonyl carbon of the acetoxy is not present in compound** 2**, which confirms the structure of compound** 2** to be the same as compound** 1** with the replacement of the acetoxy group and the sugar moiety with hydroxyl groups. Accordingly, the structure of compound** 2** was elucidated to be 3,3′,4′-trihydroxy-(3′′,4′′-dihydro-3′′,4′′-dihydroxy)-2′′,2′′-dimethylpyrano-(5′′,6′′:7,8)-flavone.

Cytotoxic activity evaluating models provide effective preliminary data to help in selecting plant extracts with potential antineoplastic properties for future work [30]. In view of the present data, Cytotoxic activity of the isolated flavonoids was evaluated by the MTT assay. As shown in Table 2, the pyranoflavones was shown to exhibit a potent cytotoxic activity against the three cancer cell lines while the flavonoid glycosides (**2**,**3**) showed a weak activity. This indicated that the cyclization of the oxygen atom at C-7 to form the pyran ring increases the cytotoxic activity of the flavone. Compound** 2** was the most potent cytotoxic agent against HeLa cells with an IC_50_ 0.079 *μ*M. Centaureidin (**5**) showed a very strong inhibition against HepG2 and MCF-7 cells with an IC_50_ 0.25 and 0.14, respectively.

## 4. Conclusion

In the present study, the cytotoxicity of ten flavonoids (**1**–**10**) isolated from* C. scoparia* was evaluated by the MTT assay against three human cancer cell lines. All the tested compounds displayed a different inhibition in a dose-dependent manner. Compound** 2** was the best cytotoxic agent against HeLa cell line with an IC_50_ 0.079 *μ*M, while Centaureidin (**5**) is a better cytotoxic agent against HepG2 and MCF-7 cells with an IC_50_ 0.25 and 0.14, respectively.

## Figures and Tables

**Table 1:** ^
1^H, ^13^C, and selected HMBC NMR data for compounds **1** and 2*.

Position	Compound **1**			Compound **2**		
	*δ*_H_ (*J* in Hz)	*δ*_C_	HMBC	*δ*_H_ (*J* in Hz)	*δ*_C_	HMBC
2		156.6			154.2	
3		133. 7			141.2	
4		178.2			173.7	
5	7.51 (1H, *d*, *J* = 8.9)	128.1	4, 7, 8a	7.55 (1H, *d*, *J* = 8.9)	127.9	4, 7, 8a
6	6.95 (1H, *d*, *J* = 8.9)	116.9	7, 8, 4a	6.97 (1H, *d*, *J* = 8.9)	116.5	7, 8, 4a
7		158.6			158.2	
8		108.4			107.7	
8a		155.7			155.1	
4a		118.9			117.8	
1′		129.7			129.6	
2′	7.11 (1H, *d*, *J* = 1.8)	119.5	1′, 3′, 4′, 6′	7.13 (1H, *d*, *J* = 1.8)	119.4	1′, 3′, 4′, 6′
3′		152.4			152.4	
4′		149.7			149.9	
5′	6.90 (1H, *d*, *J* = 8.1)	123.1	1′, 3′, 4′, 6′	6.93 (1H, *d*, *J* = 8.1)	123.2	1′, 3′, 4′, 6′
6′	7.01 (1H, *dd*, *J* = 8.1, 1.8)	121.2	1′, 5′	7.05 (1H, *dd*, *J* = 8.1, 1.8)	121.1	1′, 5′
2′′		79.6			78.3	
3′′	3.95 (1H, *d*, *J* = 4.8)	69.7	4′′, 8, Me^1^-2′′, Me^2^-2′′	3.81 (1H, *d*, *J* = 4.8)	69.4	4′′, 8, Me^1^-2′′, Me^2^-2′′
4′′	6.55 (1H, *d*, *J* = 4.8)	63.5	2′′, 3′′, 7, 8, 8a, CO of AcO-4′′	5.24 (1H, *d*, *J* = 4.8)	59.3	2′′, 3′′, 7, 8, 8a
Me^1^-2′′	1.42 (3H, s)	26.6	2′′, 3′′	1.41 (3H, s)	24.1	2′′, 3′′
Me^2^-2′′	1.39 (3H, s)	21.5	2′′, 3′′	1.38 (3H, s)	21.2	2′′, 3′′
OAc-4′′	1.95 (3H, s)	171/20.7				
D-Glu						
1′′′	4.95 (1H, *d*, *J* = 6.9)	102.4	3			
2′′′	3.24 (*m*)	74.8	3′′′			
3′′′	3.05 (*m*)	77.9	2′′′, 4′′′			
4′′′	3.11 (*m*)	70.1	3′′′, 5′′′			
5′′′	3.22 (*m*)	76.7	4′′′			
6′′′	3.45 (2H, *m*)	61.3				

*Measured in DMSO-*d*_6_ at 500 MHz (^1^H) and 125 MHz (^13^C).

**Table 2:** Cytotoxicity (IC_50_ in *μ*M)^a^ of compounds **1**–**10 **isolated *C. scoparia*.

IC_50_ (*μ*M)			
Cell lines Compound	HeLa	MCF-7	HepG2
**1**	3.21 ± 0.21	4.23 ± 0.11	3.91 ± 0.54
**2**	0.079 ± 0.03	0.32 ± 0.08	0.64 ± 0.12
**3 **	11.98 ± 1.79	12.21 ± 1.81	13.39 ± 1.3
**4 **	13.54 ± 2.1	12.96 ± 1.57	14.24 ± 1.21
**5 **	0.11 ± 0.02	0.14 ± 0.04	0.25 ± 0.05
**6 **	8.14 ± 1.28	6.52 ± 1.40	4.4 ± 0.50
**7**	9.31 ± 0.91	11.33 ± 1.12	12.41 ± 0.94
**8 **	1.12 ± 0.74	5.93 ± 1.12	4.2 ± 1.30
**9**	1.71 ± 0.18	7.75 ± 1.31	5.7 ± 0.85
**10**	3.54 ± 1.89	9.98 ± 1.89	7.14 ± 1.97
Deguelin	6.21 ± 0.82	5.17 ± 0.94	0.65 ± 0.11

^a^The data shown are means ± SD obtained from three independent experiments.
